# Ownership of small livestock species, but not aggregate livestock, is associated with an increased risk of anemia among children in Ethiopia: A propensity score matching analysis

**DOI:** 10.1002/fsn3.3474

**Published:** 2023-06-07

**Authors:** Taddese Alemu Zerfu, Amare Abera, Alan Duncan, Isabelle Baltenweck, Geraldine McNeill

**Affiliations:** ^1^ International Food Policy Research Institute (FPRI) Addis Ababa Ethiopia; ^2^ Global Academy of Agriculture and Food Systems Royal (Dick) School of Veterinary Studies, University of Edinburgh (UoE)‐Easter Bush Campus Roslin UK; ^3^ International Livestock Research Institute (ILRI) Nairobi Kenya; ^4^ College of Medicine and Health Sciences Wollo University Dessie Ethiopia

**Keywords:** anemia, cattle, children, goat, livestock, poultry

## Abstract

Consumption of animal source foods, through livestock production, improves children's growth and micronutrient status. However, research on the relationship between livestock ownership and childhood anemia has produced conflicting results. The current study used robust analytical approaches to examine the effect of household livestock ownership on children's anemia using the most recent secondary data from the national demographic and health survey. We followed a 1:1 closest neighborhood propensity score matching analysis. A propensity score was generated using the binary logistic regression model to compute the probability of owning livestock. From a total of 18,008 households enrolled in the latest Ethiopian Demographic and Health Survey (EDHS 2016), data of 721 index children aged 6–59 months from households owning livestock were matched with a comparable number (721) of children from households without livestock. The paired and independent t‐test, matched relative risk (RR), and standardized mean differences were used to compare the distributions of hemoglobin concentration and anemia risks between treatment and control groups. Anemia was found in more than half (54.1% and 58.8%) of children aged 6–59 months from livestock‐owning and nonowning families, respectively (*p* > .05). Aggregate ownership of livestock was not associated with hemoglobin concentration or anemia status (RR = 0.95, 95% confidence interval [95% CI] [0.87–1.04]). Species‐wise, poultry was associated with a lower (RR = 0.88, 95% CI [0.84–0.95]) anemia risk, while ownership of goat/sheep was associated with higher (RR = 1.10, 95% CI [1.03–1.17]) risk. In conclusion, ownership of small livestock species (sheep/goats and poultry), but not aggregate livestock ownership, was associated with the risk of anemia among children in Ethiopia. Therefore, agriculture‐sensitive nutrition, with a One Health lens approach, is recommended to mitigate the high burden of anemia among children in Ethiopia. In the future, a well‐controlled interventional study with more extended periods may be required to fully understand the effects of livestock production and highlight the differences seen across livestock species.

## Graphical abstract

The study aimed to examine whether the risk of anemia in young children among households owning livestock varies by livestock type or not. A 1:1 closest neighborhood propensity score matching analysis was applied. We found that aggregate livestock ownership was not associated with the risk of anemia in children. However, ownership of poultry and goats was associated with a lower and higher risk of childhood anemia, respectively.

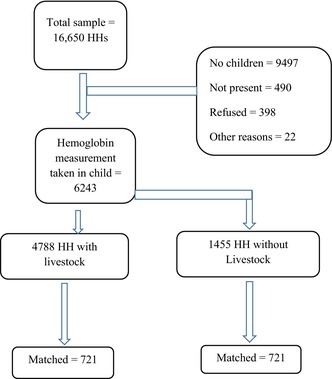

## 1 INTRODUCTION

According to the 2020 Global Nutrition Report, no country is “on course” to reach the 50% reduction target for anemia, based on country‐level progress toward the 2025 global nutrition target (Chawla, 2020). In 2019 alone, some 1.74 billion people worldwide were affected by anemia, with the highest prevalence of 39.7% observed among under 5‐year‐old children (Gardner & Kassebaum, 2020; Kassebaum, 2016). Micronutrient deficiencies (iron, vitamin B12, folate, and vitamin A) are the leading causes of anemia in low‐ and middle‐income countries (LMICs), accounting for nearly half of the global burden (Bailey et al., 2015; Martorell & Trowbridge, 2002a; Van, 2003). In Ethiopia, based on the latest Demographic and Health Survey (DHS, 2016) estimate, the prevalence of anemia among children under 5 was 57%, of which 72% were infants and young children aged 6–23 months (Gardner & Kassebaum, 2020).

Anemia in children has several long‐term consequences, leading to impaired cognitive function, diminished school performance, and retarded growth, among many others (Camaschella, 2015; Darnton‐Hill & Mkparu, 2015; Gashu et al., 2016). According to mounting evidence, iron deficiency limits psychomotor development in children, affects emotional functioning, and hampers normal physiological functions in the body (Gashu et al., 2016; Pala et al., 2010).

Several strategies and approaches have been tested and evaluated to end anemia and its sequelae among vulnerable population groups, including micronutrient supplementation, parasitic infection control, promotion of key dietary behaviors, food fortification, increasing availability and affordability of micronutrient‐rich foods (through household food production), and agricultural diversification (Kapil et al., 2019; Martorell & Trowbridge, 2002b). Studies have shown that having access to meat and milk through livestock ownership improves the growth and micronutrient status of children (Dror & Allen, 2011; Hossain & Khan, 2020; Neumann et al., 2014).

However, studies on the role of livestock ownership and child anemia have yielded conflicting findings. For example, in some studies (Christian et al., 2019; Jones et al., 2018), aggregate livestock ownership was associated with higher odds of child anemia; while another study (Lambrecht et al., 2021) with a lower risk (Lambrecht et al., 2021). In Asian countries like Bangladesh, Cambodia, Nepal, and Philippines, a homestead food production model that included animal production improved household food security and lowered the risk of anemia by improving nutrient density (Talukder et al., 2010).

Conversely, theoretical studies have also shown that livestock keeping may lead to infection (Cash‐Goldwasser et al., 2018; Raghunathan et al., 2005; Zerfu et al., 2022), and that infection and inflammation contribute to anemia (Hasyim et al., 2018; LaBeaud et al., 2015; Lambrecht et al., 2022). However, there is insufficient evidence to conclude whether livestock production improves, or remains unrelated to childhood anemia in LMIC settings (Lambrecht et al., 2019), including Ethiopia (Abdurahman & Id, 2021; Omer & Hailu, 2023; Passarelli et al., 2020). Furthermore, existing evidence on the relationship between livestock ownership and its health consequences is mainly based on observational studies that are short of causal‐effect association. There is also a systematic difference in the risk of anemia between children from livestock‐owning and nonowning households, necessitating the separation of the “true” effect of ownership (“causal effect”) from the effect of initial differences in the characteristics of the two groups (“selection effect”). The “classical analytic problem” is that this counterfactual situation cannot be observed in the real world. To that end, we used a quasi‐experimental approach—propensity score matching (PSM) analysis method, which has been widely used in observational studies to reduce confounding biases.

PSM, introduced in 1983 by Paul R. Rosenbaum and Donald Rubin (Rosenbaum & Rubin, 2006), is a statistical matching technique used in the analysis of observational data to estimate the effect of a treatment, policy, or other intervention by accounting for the covariates that predict receiving the treatment. Particularly, it helps to eliminate bias caused by confounding variables detected in a treatment effect estimate produced by simply comparing outcomes between units with treatment and those that did not.

The purpose of this study was to address these issues using a semiparametric matching technique. We specifically examined the effect of household livestock ownership on children's anemia using robust analytical approaches based on the most recent (2016) secondary data from the Ethiopian demographic and health survey (EDHS).

## 2 METHODS

### 2.1 Data source

The dataset used in this analysis was obtained from the MEASURE DHS database at http://dhsprogram.com/data/ for the 2016 EDHS. The 2016 survey is the fourth comprehensive survey in this panel survey. Use of data followed approval from the DHS program office. The data were collected from January 18 to June 27, 2016 using trained and experienced data collectors. The survey used the standard MEASURE DHS questionnaire adapted to the Ethiopian context.

### 2.2 Sampling procedures and sample size

**FIGURE 1:**
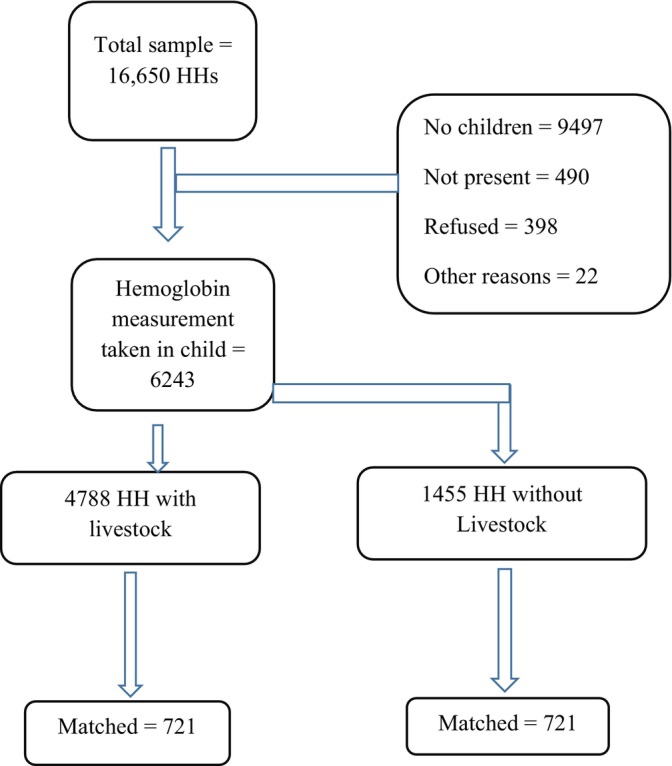
Flowchart for selection of children from HH who do any livestock.

The 2016 EDHS sample was stratified and selected in two stages. In the first stage, a total of 645 clusters (202 in urban areas and 443 in rural areas) were selected based on the 2007 population housing census. A household listing was carried out in all of the selected clusters from September to December 2015, which served as a sampling frame for the selection of households in the second stage. A total of 18,008 households were considered, of which 16,650 (92.4% of the response rate) households were eligible for analysis. From the 16,650 sampled households, 9497 households had no children aged 6–59 months, and 490 children or households were not present during data collection (Figure 1).

### 2.3 Study variables

The outcome variable for this study was hemoglobin centration (mg/dL) and the proportion of children aged 6–59 months with mild, moderate, or severe anemia or with any anemia. Blood samples for anemia testing were taken from all children aged 6–59 months whose parents or other individuals responsible for them gave consent. Blood samples were drawn from a drop of blood taken from a finger prick (or a heel prick in the case of children aged 6–11 months) and collected in a microcuvette. On‐site hemoglobin analysis was performed with a battery‐operated portable HemoCue analyzer. The readings were adjusted for altitude (Sullivan et al., 2008) and anemia was also defined according to DHS guide (Croft et al., 2018) as follows:

1. Any anemia: hemoglobin count is less than 11 g/dL or 110 g/L.
2. Mild anemia: hemoglobin count is between 10.0 and 10.9 g/dL or 100–109 g/L.
3. Moderate anemia: hemoglobin count is between 7.0 and 9.9 g/dL or 70–99 g/L.
4. Severe anemia: hemoglobin count is less than 7.0 g/dL or less than 70 g/L.

The denominator was the number of children between 6 and 59 months who were measured in households selected for anemia testing and who stayed in the household on the night before the survey. Livestock ownership was defined as any livestock presence in the household, it was a “yes” or “no” question. Specific livestock ownership was also defined. For example, sheep ownership indicates the absence or presence of sheep in the household. For any type of livestock, the presence of one or more animals indicates ownership.

### 2.4 Matching procedures

A propensity score was generated using the binary logistic regression model to compute the probability of owning livestock. The variables selected as covariates related to the outcome include place of residence (urban vs. rural); sex of household head (male‐headed household vs. female‐headed household); ownership of usable land or farm (no vs. yes); maternal age; maternal body mass index (BMI); maternal anemia; child age in months; child anthropometrics (height for age, weight for age, and weight for height); maternal education; number of under five children in the household; type of toilet facility (improved or unimproved), and wealth tertile (Brookhart et al., 2006).

After generating the score, nearest neighbor matching was used with a caliper of 0.01 and 1:1 ratio of treatment and controls. Using the household livestock ownership variable (HV246), 721 livestock‐owning households were matched with 721 households without livestock giving a total sample of 1442 households. Specific livestock ownership (cow, goat, etc.) was also matched with different sample sizes (Figure 1 and Table 1). Statistical significance was declared using a *p‐*value less than or equal to .05 for all analyses.

**TABLE 1:** Sample size for livestock ownership and anemia, after propensity score matching.

Livestock ownership	No^a^	Yes	Match found for^b^	Total sample
Cattle	4239	2004	1674	3348
Cow	2819	3424	1566	3132
Sheep	4015	2228	1563	3126
Goat	4497	1746	1655	3310
Chicken	3601	2642	1780	3560
Any livestock	1455	4788	721	1442

^a^ “No” represents control group and “yes” represents treatment groups.

^b^ Since 1:1 matching was applied the sample size for treatment and control groups are identical.

### 2.5 Statistical analysis

The data were downloaded from measure DHS as SPSS file, cleaned, and exported to STATA/MP version 16.0 for analysis. We used the household weight (hv005) to deal with the survey weights in the PSM which is the inverse of its household selection probability multiplied by the inverse of the household response rate in the stratum. The matched frequencies and percentages were calculated.

The mean and mean differences in hemoglobin concentration were calculated using paired t‐test and independent *t*‐test, respectively. The association between livestock ownership and anemia was computed using chi‐square statistics. The relative risks (RR) with a 95% CI were computed using binomial regression model.

PSM was used to determine the effect of livestock ownership on child anemia. Since DHS data are cross‐sectional in nature, the inference is difficult without statistical adjustments. The PSM method allows for the design and analysis of observational data while accounting for the randomization issue via baseline variables (Austin, 2011). The propensity score is thus the probability of treatment assignment conditional on observed baseline characteristics.

## 3 RESULTS

### 3.1 Background characteristics

From a total of 18,008 households enrolled in the study, 1442 index children aged 6–59 months from a similar number of households formed the sample to evaluate the effect of overall livestock ownership on child anemia. The mean propensity score ± standard deviation for the total sample was 0.67 ± 0.27, while it was 0.67 ± 0.26 for the treatment group and 0.68 ± 0.27 for the control group. The mean age of children was 35.2 ± 15.3 months, and 50.6% of the children were males.

After PSM, the comparison of treatment and control groups showed no difference based on the baseline variables. The variables included sex of children, mean age of children, child anthropometrics, maternal age, maternal BMI, number of under five children in a household, sex of household head, place of residence and ownership, and usable land or farm. This was based on the relevance and adequacy of the variables and samples for analysis. We eliminated some of the original variables since they were not significant, as this greatly reduced the sample size. So, to have large enough samples, we cautiously dropped those variables which had low influence in the matching. In all these variables, there was no significant difference between the two groups (*p*–value > .05) in any of the cases, as shown in Table 2.

**TABLE 2:** Comparison of livestock owners (treatment) and nonowners (control) children aged 6–59 months, Ethiopia, 2016.

Variables	Ownership status		*T*‐static/chi‐square & *p*‐Value	
	Yes	No	*t*‐static/chi‐square	*p*‐Value
Male children (%)	52.84	47.29	2.84	.092
Child age in months (mean & SD)	35.02 ± 0.56	35.29 ± 0.57	0.34	.731
HFA—percentile	24.12 ± 1.15	23.82 ± 1.13	−0.189	.850
WFA—percentile	19.62 ± 0.95	17.86 ± 0.87	−1.36	.174
WFH—percentile	31.22 ± 0.99	29.37 ± 0.95	−1.35	.176
Number of under five children	1.64 ± 0.02	1.59 ± 0.02	−1.29	.196
Maternal age in years	36.96 ± 0.45	36.01 ± 0.46	−0.25	.805
Maternal BMI	21.03 ± 0.16	20.87 ± 0.14	−0.733	.463
Male‐headed households (%)	70.59	73.09	1.11	.292
Urban dwellers (%)	30.65	29.12	0.40	.527
Land owners (%)	49.93	47.85	0.624	.429

*Note*: Continuous variables are expressed as mean and SD, and categorical variables were presented as proportions.

Abbreviations: BMI, body mass index; HFA, height for age; SD, standard deviation; WFA, weight for age; WFH, weight for height.

### 3.2 Anemia status among children aged 6–59 months in Ethiopia

Anemia was found in more than half (54.1% and 58.8%) of the children from livestock‐owning and nonowning families, respectively. In contrast to households without cattle, which had the lowest prevalence of severe anemia (1.53%), children from households with a goat (4.35%), or sheep (4.61%) had the highest prevalence of severe anemia. Table 3 shows the prevalence of anemia in the treatment and control groups.

**TABLE 3:** Prevalence of anemia among treatment and control children aged 6–59 months, Ethiopia, 2016.

Livestock type	Anemia status, frequency (%)				Any anemia
	Normal	Mild	Moderate	Severe	
Cattle					
No	743 (44.38)	410 (24.49)	467 (27.90)	54 (3.23)	931 (55.62)
Yes	769 (45.94)	395 (23.60)	452 (27.00)	58 (3.46)	905 (54.06)
Sheep					
No	688 (44.02)	383 (24.50)	448 (28.66)	44 (2.82)	875 (55.98)
Yes	638 (40.82)	380 (24.31)	473 (30.26)	72 (4.61)	925 (59.18)
Goat					
No	777 (46.95)	380 (22.96)	449 (27.13)	49 (2.96)	878 (53.05)
Yes	685 (41.39)	380 (22.96)	518 (31.30)	72 (4.35)	970 (58.61)
Chicken					
No	756 (42.47)	422 (23.71)	537 (30.17)	65 (3.65)	1024 (57.53)
Yes	869 (48.62)	424 (23.82)	433 (24.33)	54 (3.03)	911 (51.18)
Livestock (any)					
No	311 (43.13)	186 (25.80)	213 (29.54)	11 (1.53)	410 (56.87)
Yes	331 (45.91)	167 (23.16)	194 (26.91)	29 (4.02)	390 (54.09)

### 3.3 The effect of ownership of different livestock species on hemoglobin concentration

Generally, there was no difference in hemoglobin concentration in children between households owning livestock and those without livestock (mean difference [MD] = −0.13 g/L, *p* > .05). The effect of ownership of different livestock species was also analyzed to determine the association with hemoglobin concentration. Cattle ownership was not associated with hemoglobin concentration among children (*p* > .05). In contrast, goat and sheep ownership were both associated with decreased children's hemoglobin concentration (MD = 2.48 g/L, *p* < .001, and MD = 1.76 g/L, *p* < .01, respectively). In contrast, poultry ownership was associated with increased children's hemoglobin concentration compared with nonowners (MD = 2.63 g/L, *p* < .001; Table 4).

**TABLE 4:** Mean, mean difference of children's hemoglobin concentration (g/L), and the association with different livestock groups in EDHS 2016.

Livestock type	Sample	Mean (SD)	*t*‐static	*p*‐Value
Any livestock				
No	771	105.9 ± 15.6	0.150	.881
Yes	771	105.8 ± 17.6		
Combined	1442	105.9 ± 16.6		
Difference (no‐yes)		−0.2		
Difference		−0.2		
Cattle				
No	1674	105.5 ± 17.2	−1.075	.282
Yes	1674	106.1 ± 17.3		
Combined	3348	105.8 ± 17.2		
Difference		−0.6		
Goat				
No	1655	106.3 ± 16.8	4.07	.000***
Yes	1655	103.8 ± 18.2		
Combined	3310	105.1 ± 17.6		
Difference		2.5		
Sheep				
No	1563	105.6 ± 16.6	2.805	.005**
Yes	1563	103.9 ± 18.4		
Combined	3126	104.7 ± 17.5		
Difference		1.8		
Chicken				
No	1780	104.6 ± 17.0	−4.59	.000***
Yes	1780	107.2 ± 17.1		
Combined	3560	105.9 ± 17.1		
Difference		−2.6		

*Note*: *p < .05; **p < .01; ***p < .001.

Abbreviation: EDHS, Ethiopian Demographic and Health Survey.

### 3.4 RR of anemia and livestock ownership

There was no difference between control and treatment groups (overall livestock ownership) in the anemia status of children, RR = 0.95, 95% CI [0.87–1.04]. Aggregate cattle ownership status of all species (including camels), RR = 0.97, 95% CI [0.91–1.03], and cows from cattle species, RR = 0.95, 95% CI [0.88–1.00], showed no significant difference in risk of anemia. However, goat ownership increased the risk of anemia by 10%, RR = 1.10, 95% CI [1.03–1.17], while ownership of poultry decreased the risk by 12%, RR = 0.88, 95% CI [0.84–0.95] (Table 5).

**TABLE 5:** Logistic regression of the RR of anemia and livestock ownership in EDHS 2016.

Livestock type	RR	95% CI
Any livestock	0.95	0.87–1.04
Cattle	0.97	0.91–1.03
Cow	0.95	0.88–1.00
Goat	1.10	1.03–1.17*
Sheep	1.05	0.99–1.12
Chicken	0.88	0.84–0.95**

Abbreviations: CI: confidence interval; EDHS, Ethiopian Demographic and Health Survey; RR: relative risk.

* *p* < .05.

** *p* < .01.

## 4 DISCUSSION

The current study aimed to assess the collective as well as species‐specific effects of livestock ownership on anemia status and hemoglobin concentration of children in Ethiopia using national data from the most recent available demographic and health survey. We applied the PSM analysis approach, which is a quasi‐experimental method with a robust statistical technique that constructs an artificial control group by matching each treated unit with a nontreated unit of similar characteristics. Generally, we found that aggregate livestock ownership was not associated with anemia in children. However, ownership of some livestock species—mainly small livestock (goats or sheep), and poultry, has been linked to risk of childhood anemia in reverse direction. Ownership of small animals (goats or sheep) was found to raise the risk of childhood anemia by 10%, while poultry ownership decreased the risk by 12%.

However; inferences from these results should be cautious, as some of the approaches and techniques used to reach to these conclusions come with several important caveats. First, because a large number of subjects were excluded from the study due to a lack of appropriate matches, which could also be considered a disadvantage of the PSM, the results may not be fully representative of the original data. Second, the types of livestock given here are based on recall and reporting by the household head, which is neither cross‐checked with the existing administrative records at community level, nor objectively verified during data collection. Falsification is possible due to underreporting and/or misreporting of household wealth in such settings. There could also be a recollection and misclassification bias issue, which could lead to an under or overestimation of the real strength of the association.

Despite the limitations mentioned above, the results of the study revealed an absence of association when aggregated, but a mixed association between livestock ownership and the risk of anemia in children when disaggregated by species. The lack of risk relationship between aggregated livestock ownership and childhood anemia could be due to the neutralizing (counterbalancing) effect of the beneficial and negative impacts of different livestock species aggregated together. It could also be linked to the total number of cattle held by households, which the current study did not assess. Furthermore, the short observation time or cross‐sectional nature of the study design may have concealed some of these effects that may have been detected using other study designs/ methods. This finding is also different from other studies that reported either beneficial (positive) (Lambrecht et al., 2021) or adverse (negative) associations (Christian et al., 2019; Jones et al., 2018) between livestock ownership and anemia risk among children in LMIC settings.

Our findings are also supported by both observational and experimental studies from LMIC settings (Michaux et al., 2019; Osei et al., 2017). For example, a Nepalese experimental study involving home gardening, poultry, and nutrition education programs found a reduced risk of anemia in children (Osei et al., 2017). An observational study (Acharya et al., 2021) conducted using data collected from 28 Sub‐Saharan African countries has also reported that livestock ownership is associated with a lower risk of hemoglobin concentrations in children.

Conversely, when disaggregated by species, consistent with others' findings, our study has also showed that poultry keeping has been repeatedly associated with improved hemoglobin concentration and lower anemia status (Jones et al., 2018; Lambrecht et al., 2019; Osei et al., 2017). But, ownership of small livestock such as sheep, and goats has been linked to a reduced hemoglobin concentration or higher anemia risks.

Consistent with our findings, other studies (Custodio et al., 2008; Jankowska et al., 2012; Omer & Hailu, 2023; Osei et al., 2017) have also found that poultry provides a protective advantage against anemia in children. Perhaps, not all studies have showed beneficial effects, as there are also some observational and experimental studies showing a reverse association or effect (Olney et al., 2009).

Our study has also highlighted that ownership of small livestock such as sheep and goat is a potential risk factor of anemia among children. This is consistent with other studies (Bailey et al., 2015; Basnet & Schneider, 2009; Tine et al., 2013; Walden et al., 2022). The main route into this could be increased parasite infestations (like hookworm) of children in households who keep small livestock like sheep and goats, which is a key risk factor for childhood anemia (Grimes et al., 2017; Stoltzfus et al., 1997) In Senegal (Walden et al., 2022), for example, a study found a persistently high prevalence of anemia in farmers and family members who owned sheep due to intestinal parasite infections during the rainy season.

Morbidities associated with goat's milk feeding to children could be another cause for goat and/or sheep ownership increasing the frequency of anemia. According to a review by Basnet and Schneider (2009), goat's milk is linked to severe electrolyte abnormalities, metabolic acidosis, megaloblastic anemia, hemolytic uremic syndrome, and infection. This indicates that children from households that keep sheep/goats and possibly consume their milk are at a higher risk of anemia.

### 4.1 Future research

While this study focused on anemia and hemoglobin concentration as main or primary outcomes, further research is needed to determine the effect of households' livestock ownership on other health and nutritional outcomes of children. In our present analysis, we showed that the observed effect is small and is likely to vary by indicator and by context. Observational studies of longer duration or well‐controlled interventional studies might be needed to elucidate additional or net effects of livestock intervention.

## 5 CONCLUSION

Although in‐depth analysis of national survey data applying a robust (PSM) method, we examined the effect of livestock ownership on anemia status of children in a resource‐limited setting—Ethiopia. We found that even though the aggregate ownership of livestock had not been associated with anemia of children, disaggregated analysis by species showed a modest (10%–12%) bidirectional effect of keeping small livestock (goat and sheep), and poultry.

As a result, households in Ethiopia may benefit (indirectly) from small‐scale poultry production to lower the risk of anemia in children. More research, employing rigorous study design (randomized trials) and/or synthesis of available evidences through systematic reviews and pooling of effect sizes, is needed before policy recommendations are made. It is also useful to know why the ownership of small livestock like sheep and goats is associated with an increased risk of childhood anemia in such settings.

## AUTHOR CONTRIBUTIONS

**Taddese Alemu Zerfu:** Conceptualization (equal); data curation (supporting); formal analysis (lead); investigation (lead); methodology (equal); project administration (lead); visualization (equal); writing – original draft (lead); writing – review and editing (equal). **Amare Abera:** Data curation (equal); formal analysis (equal); investigation (supporting); methodology (supporting); software (equal); writing – review and editing (supporting). **Alan Duncan:** Conceptualization (equal); funding acquisition (lead); investigation (equal); methodology (equal); resources (equal); supervision (lead); validation (equal); writing – review and editing (equal). **Isabelle Baltenweck:** Funding acquisition (lead); methodology (supporting); resources (equal); supervision (supporting); writing – review and editing (equal). **Geraldine McNeill:** Conceptualization (lead); data curation (equal); funding acquisition (lead); investigation (equal); methodology (equal); project administration (equal); resources (equal); supervision (lead); validation (equal); writing – review and editing (lead).

## FUNDING INFORMATION

This project has received funding from the European Union's Horizon 2020 research and innovation program under the Marie Skłodowska‐Curie grant agreement No 801215 and the University of Edinburgh Data‐Driven Innovation program, part of the Edinburgh and South East Scotland City Region Deal. The study is also supported by the International Livestock Research Institute (ILRI), and the University of Edinburgh. TAZ also used some of his time at the International Food Policy Research Institute (FPRI) in revising this work.

## CONFLICT OF INTEREST STATEMENT

The authors declare that they have no conflict of interest.

## ETHICS STATEMENT

We accessed the data after securing permission from the Measure DHS organization. Furthermore, data were collected in accordance with national and international ethical guidelines.

## CONSENT FOR PUBLICATION

Not applicable.

## Data Availability

Data are available in the public domain www.measuredhs.com.
